# Historic drought shifted fishing from a stigmatized practice and food to an adaptive livelihood among Daasanach pastoralists in northern Kenya

**DOI:** 10.21203/rs.3.rs-8845701/v1

**Published:** 2026-02-19

**Authors:** Kedir Teji Roba, Alfredo J Rojas, Matthew J. Douglass, Natalie C Meriwether, A A McGrosky, H Jacobson, Anna Tavormina, Suha Arshad, G Khosi, DR Braun, R Nzunza Nzunza, E Ndiema, H Pontzer, Asher Y. Rosinger

**Affiliations:** Haramaya University; Pennsylvania State University; University of Nebraska–Lincoln; Pennsylvania State University; Elon University; Northwestern University; Duke University; Vanderbilt University; National Museums of Kenya; George Washington University; Kenya Medical Research Institute; National Museums of Kenya; Duke University; Pennsylvania State University

**Keywords:** Fishing, food insecurity, drought, stigma, resilience

## Abstract

**Introduction:**

Droughts disrupt livelihoods, forcing populations to adapt. This study examines how drought-driven livelihood stress is shifting Daasanach pastoralists’ perceptions of and engagement with once stigmatized practices—fishing and eating fish—in northern Kenya.

**Methods:**

This study used mixed methods within the Daasanach Human Biology Project to assess variations in fish consumption and dietary patterns across pre-drought, drought, and post-drought periods. Qualitative data gathered through six focus group discussions (FGDs) provided insight into experiences of food insecurity, coping strategies, and livelihood adjustments, thereby enriching the interpretation of previously collected quantitative trends in fish consumption.

**Results:**

Between 2019 and 2024, the onset of a historic drought led to increased fish consumption that persisted post-drought. Findings reveal four interrelated themes. First, fishing changed from a stigmatized coping strategy associated with poverty to a respected livelihood conferring social status. Second, food insecurity catalyzed diversification in food culture, with fish becoming an accepted staple protein following widespread livestock loss. Third, fishing functioned as a pathway to economic and social adaptation, enabling households to purchase food, rebuild assets, support children’s education, and enhance women’s economic autonomy. Finally, this adaptation carried unintended consequences, including crocodile attacks, challenges related to fishing equipment and markets, and water-related illnesses.

**Conclusion:**

Fishing, initially adopted as a temporary coping mechanism, has become an integral livelihood due to the drought. Diversifying livelihoods can mitigate food insecurity and enhance resilience among pastoralists confronting climate change.

## Introduction

Climate change is a pressing global issue that disproportionately affects indigenous and marginalized populations (Rahman & Alam, 2016). The resilience of social-ecological systems is critical for communities, particularly in developing countries, to adapt effectively and address future challenges (Change, 2014; Toulmin, 2009). The Intergovernmental Panel on Climate Change (IPCC) notes that pastoralist communities in developing countries are especially vulnerable to climate variability (Change, 2014; Uddin, Bokelmann, & Entsminger, 2014). In East Africa, recurrent droughts have increasingly threatened pastoralist livelihoods, attracting attention from researchers and policymakers (Aalders, Bachmann, Knutsson, & Musembi Kilaka, 2021; Galaty, 2013; Lind, Okenwa, & Scoones, 2020). Over 265 million people in the Horn of Africa, including pastoralists and agro-pastoralists, depend primarily on livestock, yet they face pressures from land loss, economic inequality, and climate change (Galaty, 2013; Knutsson, Mureithi, Wredle, & Nyberg, 2021; McPeak, Little, & Doss, 2011).

Severe droughts lead to food and water shortages, economic instability, and social disruption, which increases psychological stress in those affected. The interruption of established food systems leads to both immediate and long-term modifications in dietary habits and sources of nutrition (Dlamini, Mtintsilana, Craig, Mapanga, & Norris, 2024; Marshall & Wrangham, 2007). Short-term responses, or *coping strategies*, are reactive measures used to manage sudden shocks, typically triggered by rapid-onset events such as floods or brief droughts, whereas longer-term behavioral changes aimed at adjusting to recurring or prolonged conditions are considered *adaptations* (Davies & Hossain, 1997). During times of food scarcity, communities often resort to consuming less preferred and lower-cost food options out of necessity (Dlamini et al., 2024; Marshall & Wrangham, 2007). Foraging for wild foods is one coping strategy that enhances physical access to nutrition during periods of insecurity. According to the Food and Agriculture Organization (FAO), wild foods include uncultivated or undomesticated plants (e.g., tubers, roots, vegetables, leaves, fruits) and animals (e.g., insects, amphibians, birds, small mammals, reptiles) gathered for consumption (Heywood, 1999). Research conducted in Tanzania, Ethiopia, and Bangladesh indicates that gathering wild fruits, plants, and small freshwater fish is crucial when traditional food sources are inadequate (Dlamini et al., 2024; Fentahun & Hager, 2009). These dietary adjustments diversify nutritional intake but may involve foods that are culturally less desirable or nutritionally insufficient. Coping strategies should therefore be regarded as a continuum of responses influenced by available livelihood assets and resource (Dlamini et al., 2024; Fentahun & Hager, 2009).

Adaptation responses, in contrast, are generally planned and often collective, managing gradual changes or increasing climatic intensity (Boyd et al., 2008; Davies & Hossain, 1997). Local-level adaptations are often reactive, while institutional interventions involve anticipatory planning through policies and programs (Burton, Soussan, & Hammill, 2003). Adaptation implies goal-oriented, sustained action to anticipate threats or mitigate impacts, leading to longer-term behavioral change. Smit and Skinner (Smit & Skinner, 2002) propose a typology of adaptation based on intent, duration, scale, and form, distinguishing autonomous responses from planned strategies (Smit, Burton, Klein, & Wandel, 2000).

Strengthening the resilience of social-ecological systems enables communities to respond to future climate change (Toulmin, 2009). Adaptation and building adaptive capacity are key to sustainable, equitable development in climate-sensitive regions (Africa, 2005; Remling & Persson, 2015). Adaptation draws on multiple forms of capital—natural, social, human, and financial—and individuals employ asset-, consumption-, and income-based coping strategies depending on available resources (Adger, Arnell, & Tompkins, 2005; Adger et al., 2009).

The prolonged drought in the Greater Horn of Africa from October 2020 to April 2023 caused severe water and food shortages, affecting approximately 51 million people—including 5.4 million in Kenya (WHO, 2023). Large-scale food insecurity during this period altered the traditional diets and livelihood practices of many pastoral communities, such as Daasanach semi-nomadic pastoralists living in northern Kenya, who experienced extensive livestock losses. During the drought it was observed that many community members began incorporating fish (*bee*) into their diets challenging the long-held stigma of fish as “the food of the poor” (*Guom gal he mana*) (Almagor, 1987; Houtteman, 2011).

Traditionally, fishing was limited to the Dies (/di s/) and members of the Riele community (Houtteman, 2011). Pastoralists typically did not catch fish themselves but obtained it through exchange with their Dies friends. In a study conducted by Almagor (Almagor, 1987), when asked why Daasanach ate fish despite believing it could harm their livestock, pastoralists explained that refusing a gift would be socially offensive. As one respondent noted, accepting the fish was necessary to avoid insulting their hosts (ibid.). Thus, their consumption of fish was traditionally motivated less by personal preference and more by the need to maintain important social relationships (Almagor, 1987). However, Sagawa (Sagawa, 2021) observes that since around 2010, fish consumption among the Daasanach has begun to shift from a socially symbolic practice to a more integral part of economic life. Environmental pressures such as drought, livestock loss, and climate variability have prompted some community members to adopt alternative livelihoods, with fishing becoming an important source of both food and income, particularly near Lake Turkana. More research is needed to understand how extreme climatic events act as shocks that shift cultural preferences among pastoralists and affect coping strategies used to mitigate food insecurity.

**Figure 1:**
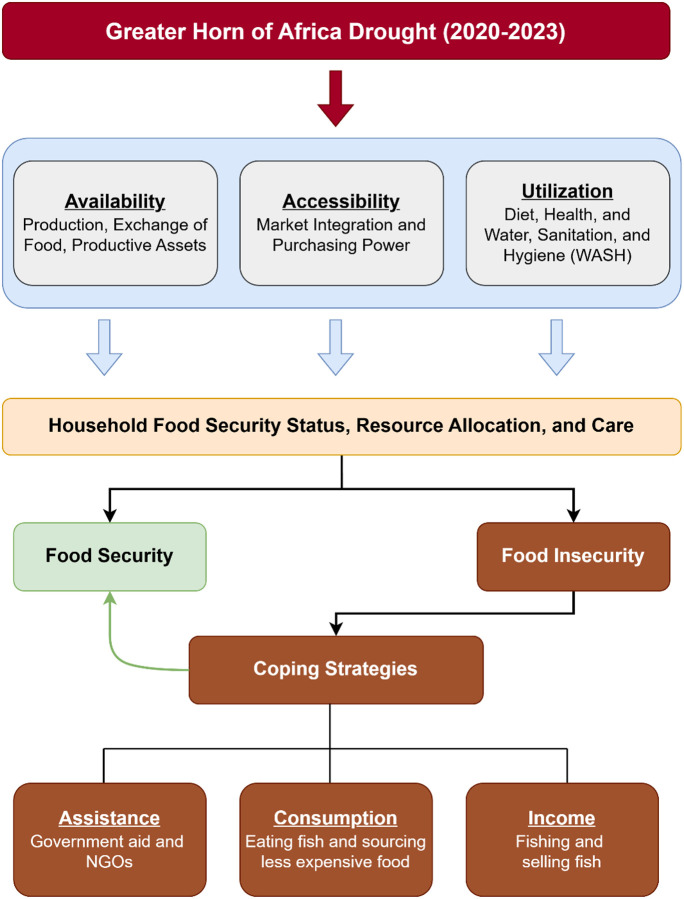
Conceptual framework demonstrating how food insecurity induced by drought affects coping practices of Daasanach communities in northern Kenya.

This study utilizes data collected over the course of an extreme climatic event to investigate the impact of severe drought on pastoralist coping strategies, with particular emphasis on fish consumption and fishing practices and its impact on food insecurity. An explanatory sequential mixed-methods design was employed, where quantitative data were collected and analyzed first, followed by qualitative data to contextualize and explain the findings. The primary research question this study examines is how drought-induced changes influenced livelihood diversification and dietary practices among Daasanach semi-nomadic pastoralists between 2019–2024. Focus group discussions were conducted to assess how attitudes toward fishing and fish consumption—originally subject to cultural stigma—shifted from being short-term coping mechanisms to long-term adaptive strategies integrated into household livelihoods. Figure 1 illustrates a conceptual framework that traces the pathways of the effects of the historic drought on the availability, accessibility, and utilization of food and impact on food security. When food insecurity increases, populations must resort to coping strategies to survive, including reliance on food aid, changing consumption patterns that range from minimizing the portion size of food to eating wild food, or finding alternative income sources from local sources. Studies indicate that some of these coping strategies may eventually become adaptive strategies (Folke, 2006; Folke et al., 2010)

## Methods and materials

### Study Area and Population

This study was undertaken in Marsabit County, Kenya among Daasanach communities. Daasanach reside in arid and semi-arid regions of northern Kenya and southwestern Ethiopia, including the northeastern shores of Lake Turkana, the lower Omo River Valley, and its delta (Gebre, 2012; Mwamidi et al., 2018). An estimated 19,000 individuals live in northern Kenya’s hot, semi-arid desert, distributed across 26 villages on the northeastern edge of Lake Turkana near the Ethiopian border (KNBS, 2019). Traditionally, livestock, such as cattle, goats, and camels, underpin Daasanach economy, diet, and rituals, providing milk, meat, and other products that serve as both nutritional staples and markers of social status (Houtteman, 2011; Kiura, 2005). Sorghum and maize supplement their diet, usually as porridge or fermented beverages during daily meals and ritual gatherings. Despite living on the shores of Lake Turkana, fishing was not historically a component of Daasanach livelihoods. Fish, considered a low-status food, were typically consumed by households with limited or nonexistent livestock wealth or during periods of severe and widespread scarcity (Houtteman, 2011). Uptake of fishing increased slightly after 2013 and rose sharply after 2020, following the Greater Horn of Africa drought (Rosinger et al., in review). Further information about the study area and the Daasanach community can be found in previous publications (Bethancourt et al., 2023; Ford et al., 2023; Rosinger et al., 2021). This paper utilizes data derived from the Daasanach Human Biology Study, gathered during several time periods: prior to the drought in 2019, at the peak of the drought in 2022, end of the drought in 2023, and one-year post-drought in 2024. The qualitative component was carried out in the summer of 2024, after the drought.

### Study Design

An explanatory sequential mixed-methods design was employed. The quantitative component of the Daasanach Human Biology Project assessed longitudinal trends in household food security, weekly fish consumption, and dietary practices before, during, and after the drought. In brief, household food insecurity was measured using the Household Food Insecurity Access Scale (HFIAS) (Coates, Swindale, & Bilinsky, 2006), translated into the Daasanach language. Following the quantitative data, qualitative data were collected to explore lived experiences of food insecurity and coping strategies. It is the qualitative data we present in this paper. Qualitative data collection relied on a phenomenological approach to understanding participants lived experiences and the meanings they attach to these experiences. Focus group discussions (FGDs) facilitated in-depth dialogue which enabled the emergence of unanticipated insights and allowed the comparison of reported versus actual behaviors. These qualitative insights complemented the quantitative findings, which indicated increased fish consumption post-drought (Moustakas, 1994). Study tools were designed by KTR and AYR.

### Sample size and sampling

Surveys took place at the same time of year in each study wave across the four study waves in 2019, 2022–2024 and is described in detail elsewhere (Rosinger et al., under review; Meriwether et al., under review). Briefly, every third household was sampled in 7 communities with an 8th community added in 2023. The total sample includes 965 observations from 513 adult Daasanach semi-nomadic pastoralists, followed across the study period.

In 2024, following the fourth survey wave, we conducted six FGDs—three male and three female groups—comprising 40 participants (20 men, 20 women) from two communities: one community close to lake Turkana (~ 2 km) and one farther away (~ 8 km) following guidelines of 4–8 FGDs needed to reach saturation of themes (Hennink & Kaiser, 2022). Participants for FGDs were drawn from the longitudinal survey cohort with an equal sex breakdown in the sample.

### Data Collection

Prior to the FGDs, the questions and guides were piloted with six local community members to ensure cultural and linguistic appropriateness, and revisions were made based on feedback to improve clarity, flow, and question sequencing. Before each session, interviewers explained the study purpose, participant roles, rights, risks, benefits, and confidentiality measures. FGDs were guided by questions surrounding food security, fishing practices, fish consumption, and livelihood diversification (**Supplemental Table 1**). Female interviewers facilitated female FGDs, and male interviewers facilitated male FGDs. Each FGD included 7–10 participants and lasted 50–120 minutes. All sessions were audio-recorded, manually transcribed verbatim, and translated into English.

### Qualitative Data Analysis

Thematic analysis was used to examine the FGD data, identifying patterns in coping strategies, dietary diversification, and livelihood adaptation. Open coding was applied to break data into discrete units, followed by axial coding to group codes into broader themes. Analysis combined inductive identification of emergent patterns with deductive guidance from research objectives (Kiger & Varpio, 2020).

A six-step thematic analysis process was employed (Kiger & Varpio, 2020; Terry, Hayfield, Clarke, & Braun, 2017): (1) familiarization with the data; (2) generation of initial codes; (3) searching for themes; (4) reviewing themes; (5) defining and naming themes; and (6) producing the final report. Data triangulation enhanced validity by comparing male and female FGDs and cross-referencing with quantitative survey data. Iterative analysis ensured alignment with study objectives, and direct quotes were used to illustrate key themes. Trustworthiness of the data was attained through (1) immediate post-interview summaries, (2) team discussions to assess credibility, (3) tailored interview guides, (4) researcher reflexivity, and (5) ethical and transparent reporting. The Standards for Reporting Qualitative Research framework guided presentation of context, findings, and interpretation (O’Brien, Harris, Beckman, Reed, & Cook, 2014).

## Results

A total of 965 adults completed the survey across the four study waves between 2019 and 2024. The mean age of adults was 40.3 years (SD = 15.2; range: 18–83), with 42.8% male (Table 1). Food insecurity was high across the survey waves, peaking at the end of the drought in 2023 (Table 1). Despite persistently high food insecurity, households consuming fish were significantly less food insecure (Meriwether et al., under review).

Among the 40 FGD participants, 50% were female, 42.5% were aged 30–40 years, 35% were 17–30 years, 97.5% were married, and half lived in households of 5–9 members (Table 2). Four interconnected themes were identified that capture the complex dynamics of livelihood transformation and dietary adaptation among the Daasanach during and after severe drought:

1. Fishing: from survival to social status – transformation from coping strategy to a respected livelihood, including gender roles in fishing.
2. Food insecurity as a driver of diversification in food culture.
3. Fishing as a pathway to economic and social adaptation.
4. Unintended consequences of fishing related to physical health risks and mobility.

### Changes in social stigma of fishing

In 2019, at the beginning of the longitudinal survey, of the 240 adults only three adults (1.3%) reported that their main occupation was fishing, though one of the adults said he just sold fish but did not do the fishing himself. By 2024, 8.5% (25 of 318) adults said that fishing was their primary occupation with many more engaging in this activity in some capacity.

Both men and women participants reported that fishing was previously stigmatized in their communities. It was associated with negative connotations and viewed as a livelihood for outsiders, particularly among the Turkana, a neighboring pastoralist group on the west side of Lake Turkana. As mentioned earlier, they also stated they referred to fishers as “*Dies*,” meaning poor. This term also applied to specific families who survive as fishermen along the shores of Lake Turkana, such as the Here or El Molo people as one female participant explained, “*Nowadays, people who fish are seen as important because they have money and can support their families*.” Over time, perceptions of fishing as an economic activity have begun to shift, with increasing recognition of its income-generating potential.

In contrast, attitudes toward fish consumption were shaped by cultural perceptions and sensory concerns rather than economic value. Participants explained that eating fish was discouraged in the past, partly due to the strong smell associated with fish and fishers. As one female participant explained: *“Before, we did not want fishermen to come near us because they smelled like fish.”* This changing perception reflects a gradual revaluation of fish consumption has no more stigmatized.

Following the drought, attitudes toward fishing and fish consumption began to shift. Community members observed that the Turkana, despite facing similar challenges, were thriving because of fishing. A male participant explained: *“Fishing was once considered the work of the poor. However, after we lost all our livestock and were left with nothing, we turned to fishing to survive. Now, there are no boundaries —both men and women participate, and even elders send their children to the lake to fish.”*

One woman described how their household began diversifying into fishing: *“First, we borrowed fish from the fishermen. Then we learned how to cook, catch, and sell it. Now it is our basic food and source of income. Much of the earlier stigma associated with fishing has been alleviated, and fishing has become a legitimate and respected source of food, income, and resilience.”*

Fishing also appeared to reshape social status and identity. Men highlighted that fishers were now respected, wealthier, and considered desirable for marriage due to their ability to pay dowries in cash rather than livestock. One male participant shared: *“Fishers are now considered acceptable, and even desirable suitors, largely due to their ability to pay dowries in cash. I have two wives. For my first wife, I paid the dowry by selling many of my livestock. But for my second wife, I didn’t sell any animals—all the cash I used for the dowry came from selling fish.”*

### Food insecurity as a driver of diversification in food culture

Quantitative analysis revealed that fish consumption increased from 2019 to 2024 (Table 1). Any fish consumption in the prior week increased from 34.6% in 2019 to 63.6% in 2024, while the number of days eating fish increased from 1.1 days to 1.7 days.

The participants said eating fish was linked to food insecurity and early on was considered socially undesirable, often described as “eating worms” and had been stigmatized as “*guom gal he mana*” (food for the poor). Some parents said they avoided feeding fish to children due to perceived health risks saying it could lead to diarrheal illnesses. Nevertheless, participants increasingly recognized its nutritional value: *“Previously, we considered fish to be food for the poor and looked down on it. But recently, we observed that children who eat fish have better growth and body fat. Now, we understand the importance of eating fish because it is both delicious and nutritious.”* The participants said that it appeared that the children of fishers were healthier and more food-secure which further influenced these changing perceptions.

The droughts which devastated livestock herds drove this transformation. One man noted: *“The long drought forced us to find a way out of hunger… That was when we realized that fishing could be one way to survive - something important that could help us and our children get food, then eventually be able to earn some income.”* Qualitative data confirmed that fish have become a staple protein source—affordable and accessible. One participant explained: *“Since the community has gained access to fishing, the demand for meat has relatively declined, as slaughtering livestock is no longer feasible.”*

Historically, livestock provided both food and wealth, with milk and blood consumed routinely, while slaughter was more common during seasonal ritual cycles. The Dimi ceremony, one of the most symbolically significant rites of passage among the Daasanach, is held for first-born daughters and marks their transition into womanhood and eligibility for marriage, while also affirming lineage and community cohesion. These ritual periods were associated with increased livestock slaughter and communal consumption, often occurring during times of ecological constraint and reflecting pastoral strategies for managing herd size and sustaining social relations (Almagor, 1987). Without livestock, fishing helped alleviate food insecurity and prevent starvation. One male discussant remarked: *“When all the livestock died… they realized there were fish and used it as their basic food.”* Fish became a staple, particularly among communities near Lake Turkana. Geographic proximity to the lake influenced acceptance as participants in the community farther from the lake unanimously said that they still preferred to eat meat over fish.

### Fishing as a pathway to economic and social adaptation

Beyond subsistence, fishing has evolved into a key pathway for socio-economic recovery. Qualitative findings highlighted that income from fishing enabled households to purchase food, repurchase livestock, rebuild homes, and invest in children’s education: *“We bought animals we lost during drought, paid school fees, bought clothes… even constructed good houses with fish money.”*

Fishing became particularly crucial for women and their empowerment during the drought. A female participant explained: *“When the severe drought came and wiped out all the livestock, we felt lost… We saw that those who were fishing were healthy, and their children had something to eat, while ours were starving. That is when we decided to start fishing.”* Another woman further explained the economic benefits for women in this patriarchal society: *“After we started fishing, we can buy food for our children without selling our livestock or asking our husbands for money.”*

Fishing also transformed household roles. Children were described as alternating between herding livestock and fishing, increasing household income while diversifying responsibilities. One mother explained: *“Previously, our children would accompany livestock to distant grazing lands, often staying alone or with their fathers. Nowadays, some tend livestock while others go to the lake to fish.”* Income from fishing allows access to education previously unaffordable for many children. A father noted: *“Now, many children can attend school without stress, thanks to the income we get from fishing.”*

### Unintended consequences and challenges of fishing

The emergence of fishing also introduced a range of unintended challenges: reliance on fish traders, availability and cost of fishing equipment, relocating to the lake side and frequently changing their camping sites along the lake shore following fish availability, market dynamics, and physical health risks.

Participants reported several challenges associated with fishing, particularly related to market access, seasonal constraints, and fishing inputs. Foreign traders travel to the area by truck to purchase fish, which is considered a positive opportunity and part of participants’ livelihood diversification, especially for those who also keep livestock. This access is easier during the dry season; however, it is also when fish prices are low. Therefore, participants said they have little bargaining power and transporting fish to central markets is often not feasible. Consequently, fishers are forced to sell their catch at the prices set by the traders. In some cases, traders bring cereals such as maize and beans and exchange them for fish. While this arrangement provides short-term food access, it disadvantages fishers economically. Fishing inputs, particularly nets, are another concern, as they are either unavailable or unaffordable. To cope, some foreign traders lend or rent nets to fishers and later collect the fish at very low prices, reducing fishers’ income and independence. The limited availability of proper fishing gear—especially quality nets—forced many fishers to depend on substandard tools. As one participant noted, *“Sometimes we are forced to rent nets because we can’t afford to buy them. That affects our income from fishing.”* Seasonal constraints exacerbate these challenges: during the rainy season, trucks are often unable to reach the area, and much of the harvested fish is damaged or spoils due to limited storage and market access. The lack of proper storage for fish drying is a challenge during the rainy season. A male participant described the dilemma succinctly: *“During the rainy season, we catch a lot, but they spoil quickly because we can’t dry them properly.”* Together, these challenges highlight the vulnerability of fishers and the critical need for improved market access, input availability, and storage solutions.

Fishing dynamics led to new mobility patterns, with families temporarily relocating to fishing areas close to the lake. While Daasanach have long practiced mobility with livestock, including moving herds to eat grasses near the lake, camping for prolonged time near Lake Turkana can lead to increased exposures. One participant remarked, *“We stay near the lake for a week or month with our fishing gear and return home after catching enough.”* These extended stays—especially by children sent to fish—raised concerns about fatigue and declining health.

Finally, fishing activities expose community members to significant physical and health risks, highlighting the hazardous nature of this livelihood. Most notably, an increase in crocodile attacks occurred during this time. Crocodile attacks have led to multiple injuries and fatalities. One participant stated, *“Our [Nangolei] community has lost five people killed by crocodiles.”* In the larger longitudinal survey, our census records have shown deaths due to crocodile attacks among men and children. Weather-related dangers, such as strong winds and unstable canoes, further jeopardize fisher safety, especially during extended fishing trips. Additionally, the quality of lake water poses health concerns; a female participant remarked, *“Sometimes the lake water is dirty… has foam… people drink it and get stomach problems.”* Women also emphasized the risks fishing poses to youth, stating, *“The boys we send to fish suffer joint pain from cold water, skin rashes, and some people are even eaten by crocodiles.”*

## Discussion

This study demonstrates that fishing practices and fish consumption in the Daasanach community of northern Kenya were strongly influenced by the historic drought and related food insecurity and livelihood losses. These practices reflect more than individual choice or awareness; they emerge at the intersection of climatic vulnerability, limited economic opportunities, and the pressures of caring for family. The results underscore how changes in social attitudes drove changes in dietary practices and economic strategies. As a result, these changes have collectively strengthened the Daasanach capacity to cope with drought and livelihood loss but have also come with new challenges and risks.

### From stigma to social acceptance

The transition of fishing from a stigmatized activity to a socially accepted and valued livelihood among Daasanach illustrates key principles from livelihood diversification, resilience, and socio-ecological systems theories. Initially adopted as a coping strategy in response to recurrent droughts, livestock loss, and food insecurity, fishing reflects households’ efforts to diversify income and food sources to reduce vulnerability. The social redefinition of fishing—from “food for the poor” to a respected livelihood—aligns with stigma and social identity theories by Goffman (Goffman, 2009). Social identities are negotiated and activities previously marginalized can be redefined under new circumstances (Goffman, 2009). This diversification has significant implications for gender dynamics and empowerment within the community.

The concepts of resilience and adaptability are also important to understand how social-ecological systems transform during climatic shocks (Walker, Holling, Carpenter, & Kinzig, 2004). This diversification of fishing among Daasanach appears to have enhanced their adaptive capacity and resilience, thereby enabling households to buffer against shocks while sustaining economic and nutritional security (Walker et al., 2004). Similar to other pastoralist populations experiencing environmental and economic shocks (Galaty, 2013; McPeak et al., 2011), after the prolonged drought, Daasanach realized the importance of fishing, which led them to adopt these practices which further transformed social norms.

Importantly, women participating in fish processing and trade underscores the gendered dimensions of adaptation as indicated by Kabeer (Kabeer, 1999). Daasanach women have increasingly participated in fish processing, trade, and marketing. This new reality has enhanced their economic independence, decision-making power within households, and broader social recognition of their role in sustaining livelihoods. Overall, this case study demonstrates that adaptation strategies are not only responses to environmental stress but also processes of socio-cultural innovation, illustrating the co-evolution of livelihoods, social norms, and ecological systems (Berkes, 2000).

### Fishing emerged as a critical coping strategy and livelihood transformation

Our findings indicate that fishing has evolved beyond a coping strategy into a central component of Daasanach livelihoods, promoting both economic stability and social resilience. When households lost livestock during the historic drought, income from fishing enabled them to purchase food, rebuild homes, repay debts, and invest in children’s education, demonstrating how livelihood diversification mitigates vulnerability to environmental shocks (McPeak et al., 2011). Post-drought, men, women, and even children became involved in fishing. Through the lens of the Food Security and Livelihoods Framework (TRÓCAIRE’S, 2023), fishing represents a strategic coping mechanism that evolved into an adaptive strategy, enabling the Daasanach to diversify their incomes by mobilizing natural and human capital (Sagawa, 2021). This approach helps maintain food security, generate income, and support household well-being, thereby enhancing both short-term survival and long-term recovery.

From a socio-ecological systems perspective, Folke (Folke, 2006) emphasizes the complex and non-linear nature of system dynamics, acknowledging the presence of thresholds, uncertainties, and unexpected environmental changes. Gradual changes interact with periods of rapid transformation, and such dynamics operate across various temporal and spatial scales. Over time, these insights have informed the development of adaptive management strategies to address environmental change (Folke, 2006; Folke et al., 2010). This model indicates that the integration of fishing into daily livelihood practices reflects the dynamic interplay between environmental pressures, social structures, and economic strategies. Environmental shocks, particularly drought and livestock loss, acted as catalysts for households to diversify their livelihood portfolios (Catley, Lind, & Scoones, 2013; Lind et al., 2020). This illustrates how socio-ecological systems respond to stress through adaptive social and economic behaviors, where human actions reshape both ecological interactions and social relations.

The adoption of fishing within Daasanach livelihood strategies became a model of the long-term socio-economic resilience as Daasanach indicated they would continue to fish since “the fish are always there”. The concept of resilience highlights a community’s ability to absorb and change in response to a stressor in their environment or economic situation (Folke et al., 2010). Small-scale changes within a system, like adopting different livelihood strategies, can promote its overall resilience in the face of social-ecological change (Folke et al., 2010). The Daasanach situation utilized fishing to protect themselves from a lack of food while simultaneously having the capacity to rebuild any lost capital, among other factors.

The role of fishing in rebuilding livelihoods can also be understood through the Sustainable Livelihoods Approach (DFID, 2008). According to this approach, a livelihood is sustainable when it can cope with and recover from stresses and shocks and maintain or enhance its capabilities and assets both now and in the future, without undermining the natural resource base. This concept was later revised into the Sustainable Livelihoods Framework (Natarajan, Newsham, Rigg, & Suhardiman, 2022) which recognizes that rural livelihoods are increasingly shaped by the combined pressures of climate change and globalization. In this case, fishing income was used to repurchase livestock, construct housing, and support small-scale entrepreneurship. This diversification strengthened household capital, mitigated risks associated with environmental shocks and transformed fishing from an emergency strategy into a core component of livelihood systems.

### Structural and ecological challenges faced by fishers

The fishing sector is crucial for livelihoods in many rural communities (Sagawa, 2021); however, fishers often face significant challenges, especially regarding market access and economic vulnerability. However, it is compounded by several systemic issues, such as low fish prices and lack of bargaining power, forcing fishers to sell their catch at prices dictated by traders (Kopp & Sexton, 2021; Rashid, 2020; Wouters, 2024). This scenario aligns with findings that suggest that rural livelihoods with limited bargaining power are fundamentally disadvantaged (Kopp & Sexton, 2021; Paudel Khatiwada et al., 2017). Although researchers have noted that diversified livelihoods can typically enhance resilience against seasonality and market fluctuations, the conditions for many fishers indicate a struggle against systemic inequities that limit effective income diversification (Jueseah, Knutsson, Kristofersson, & Tómasson, 2020; Wouters, 2024).

Seasonal changes exacerbate these challenges significantly (Jueseah et al., 2020). Transportation is often rendered nearly impossible due to poor infrastructure, and consequently, much of the fish caught can spoil, leading to economic losses (Habib, Ariyawardana, & Aziz, 2023; Schmitt & Kramer, 2009). Evidence indicates that poor infrastructure hinders market access, which is vital for reducing spoilage and enhancing income security (Wu, Guan, Zhang, & Xu, 2019). As studies have highlighted, enhancing the infrastructure not only facilitates market access but also improves the dynamics of agricultural supply chains, which could also benefit the fishing sector (Habib et al., 2023; Schmitt & Kramer, 2009; Wu et al., 2019).

Fishing inputs are another crucial challenge, particularly regarding the unavailability and high cost of essential items like nets. Some fishers find themselves reliant on foreign traders who provide nets on a loan or rental basis, only to later collect their fish at reduced prices (Elahi, Li, Emam, Zhu, & Krishnan, 2024). This practice undermines the economic independence of fishers. Dependency can decrease the capacity for sustainable income generation, hence perpetuating poverty among these communities (Paudel Khatiwada et al., 2017).

Fishing also carries unintended consequences for the Daasanach community. One such consequence is a drastic shift in mobility patterns driven by fishing dynamics. Families frequently relocate temporarily to areas closer to fishing hotspots in an effort to maximize their catches, often moving along the lakeshore and remaining there for extended periods (Nunan, 2010). This pattern indicates that the growth of commercial fishing around Lake Turkana has increased the risk of overexploitation of the lake’s fish stocks. As catches decline, fishers are compelled to shift fishing locations along the shoreline to obtain adequate harvests. Such behavior is likely associated with increased selection pressure resulting from overexploitation, which often drives shifts in fish life-history traits. Previous studies conducted in this area have similarly shown that heavily exploited species exhibit notable declines in key biological traits (Muehl et al., 2025).

On the other hand, this mobility can exacerbate existing public health risks, as prolonged stays in makeshift housing increase exposure to mosquitoes. This also means that some individuals are away from their families which limits access to adequate family support, compounding the physical strain associated with fishing (Nunan, 2010; Shivakoti, 2013). While staying near the lake, people are often exposed to unsanitary conditions and rely on unclean water frequently with high salinity levels as Lake Turkana is a mildly saline lake (Rosinger et al., 2021; Rosinger et al., 2025). These conditions contribute to increased illness, including joint pain and skin rashes caused by contaminated water (Harahap, Salmah, & Mahyuni, 2024; Olaoye & Ojebiyi, 2020).

Fishing activities expose community members to significant safety hazards, introducing serious physical health risks. Crocodile attacks pose a severe threat to fishers in these study sites, with multiple reported incidents of injury and fatalities. Evidence shows that fishing and swimming in crocodile habitats substantially increase the risk of such encounters (Chakanyuka & Utete, 2022; Pooley, Botha, Combrink, & Powell, 2020; Shivakoti, 2013). Weather-related dangers, particularly strong winds, further heighten the risks faced by Daasanach fishers. During long outings on the lake, unstable canoes become especially vulnerable to accidents (Murhula et al., 2025; Rasolofoson, Onyango, Awuor, Aura, & Fiorella, 2024).

### Strengths and limitations

A key strength of this study is that it is grounded in a long-term longitudinal project which has observed changes between 2019 and 2024 which included a historic drought. This design allowed for a nuanced understanding of changes in eating habits, food insecurity, and livelihood strategies—particularly fishing —among the Daasanach community prior to, during, and after the drought. The qualitative component provided in-depth insights into social perceptions, gender dynamics, and household-level adaptation strategies that would not have been captured through surveys alone. Additionally, integrating pre-existing quantitative trends with post-drought qualitative experiences strengthens the credibility of observed dietary and livelihood transformations. However, the qualitative data were collected after the drought, so participants’ perceptions before the drought were not directly captured. As a result, the analysis relies on recalled experiences of stigma and fishing, introducing the possibility of recall bias. However, participants were asked in detail about their pre-drought experiences to help reduce this bias. Further the longitudinal data from 2019 captured the stigma surrounding fishing and eating fish, lending additional validity to our qualitative results.

## Conclusion

The historic 2020–2023 Greater Horn of Africa drought led to an increased incorporation of fishing into the livelihoods of Daasanach pastoralists. Fishing began as an emergency coping mechanism and transformed into a sustainable livelihood, reshaping social identity, economic stability, and household resilience. These changes highlight how adaptive livelihood strategies, evolving food culture, and socio-economic transformation strengthened Daasanach capacity to withstand environmental and livelihood shocks. It is important to recognize that pastoralists employ a wide range of strategies to cope with the impacts of climate change and to adapt to a continuously changing environment (Tugjamba, Walkerden, & Miller, 2023). While diversifying livelihoods and herd composition is among the most common strategies, including for the Daasanach, this approach is not always feasible, accessible, or appropriate in every context. Different local conditions give rise to differentiated adaptation strategies, yet understanding general patterns of coping and adaptation remains essential for addressing shocks and evolving socio-ecological challenges. One alternative is to “opt out” of traditional pastoralism through livelihood diversification and the acquisition of new skills for survival, a strategy that often becomes more common as population density increases. Similarly, for the Daasanach community, developing a market for fish and enhancing fishing skills could provide a viable form of livelihood diversification, but it is important to note the potential risks associated with it. As climatic variability continues to increase, these context-specific strategies will be critical for sustaining pastoral livelihoods and systems into the future.

## Supplementary Material

Supplementary Files

This is a list of supplementary files associated with this preprint. Click to download.

- SupplementalTable1.docx

## Figures and Tables

**Table 1:** Descriptive characteristics of the Daasanach adult sample (N = 965) from the 2019–2024 waves of the Daasanach Human Biology Project, assessed for food insecurity and fish consumption.

variable	Mean or percent	Standard deviation
Age (years)	40.3	15.19
Male (%)	42.8%	49.5
BMI (kg/m^2^)	18.2	2.80
Percent eating fish at least once in the prior week		
2019	34.6	
2022	56.2	
2023	56.1	
2024	63.6	
Food insecurity score by yar		
2019	17.6	5.0
2022	17.6	4.0
2023	21.2	3.4
2024	16.6	3.5
Number of days eating fish a week by year		
2019	1.1	2.6
2022	1.2	1.8
2023	1.2	1.6
2024	1.7	1.9

**Table 2:** Socio-demographic characteristics, food insecurity coping strategies, and fish consumption practices among Daasanach adult FGD participants (N = 40) from the 2024 wave of the Daasanach Human Biology Project.

VARIABLE	Categories	n (%)
Sex	Male	20 (50.0)
	Female	20 (50.0)
Age	17–30	14 (35.0)
	30–40	17 (42.5)
	41–50	9 (22.5)
Marital status	Married	39 (97.5)
	widowed	1 (2.5)
Family size	1–4	7 (17.7)
	5–9	20 (50.0)
	≥ 10	13 (32.5)
